# Pattern of DAP12 Expression in Leukocytes from Both Healthy and Systemic Lupus Erythematosus Patients

**DOI:** 10.1371/journal.pone.0006264

**Published:** 2009-07-16

**Authors:** Nicolas Schleinitz, Laurent Chiche, Sophie Guia, Gaëlle Bouvier, Julie Vernier, Alexis Morice, Elisabeth Houssaint, Jean Robert Harlé, Gilles Kaplanski, Félix A. Montero-Julian, Frédéric Vély

**Affiliations:** 1 Department of Internal Medicine, CHU Conception, AP-HM, Marseille, France; 2 U631-UMR6102, INSERM-CNRS-Université de la Méditerranée, CIML, Marseille, France; 3 Beckman Coulter Immunotech, Cellular Analysis Departement, Marseille, France; 4 U601, INSERM, Nantes, France; 5 UMR608, INSERM-Université de la Méditerranée, Faculté de Pharmacie, Marseille, France; Centre de Recherche Public de la Santé (CRP-Santé), Luxembourg

## Abstract

DAP12 is an ITAM-bearing transmembrane adaptor originally identified on the surface of Natural Killer cells. A broad expression among other immune cells was later found in myeloid and lymphoid cells. However, data on DAP12 expression pattern rely only on immunoblot and microarray analysis. Here, we describe the generation and the characterization of an anti-DAP12 monoclonal antibody. Using this novel reagent, we show that DAP12 expression is restricted to innate immune cells in basal condition. Since a decreased expression of DAP12 has been suggested in NK cells of systemic lupus erythematosus patients, we have further investigated the NK cell receptor repertoire and leukocyte expression of DAP12 in these patients and no major changes were detectable when compared to controls.

## Introduction

DAP12 (DNAX Activating Protein of 12 kDa) is a transmembrane adaptor initially identified in Natural Killer cells [1], [2], [3]. This molecule is also named KARAP (Killer cell Activating Receptor-Associated Protein) or TYROBP (TYROsine kinase Binding Protein). The presence of a YxxL–(x)_7_–YxxL sequence in its intracellular tail defines it as a canonical ITAM-bearing molecule. This motif has been described in many adaptors that are crucial to expression and function of various activating immunoreceptors. After receptor ligation, the two tyrosine of the ITAM are phosphorylated and serve as high affinity docking site for the recruitment of the tandem SH2-containing protein tyrosine kinases ZAP-70 and p72^syk^, which in turn are activated and phosphorylate many critical downstream signalling components. Along this line, DAP12 participates in cytotoxicity and cytokine production of NK cells [4], [5].

Although initially described in NK cells, DAP12 expression has been reported in various hematopoietic cells such as osteoclasts, neutrophils, macrophages and dendritic cells [4]. Interestingly, some T and B cell subsets also express DAP12 under inflammatory conditions. In humans, CD4^+^ CD28^−^ T cells that express both DAP12 and activating KIR (Killer-cell Ig-like Receptor) have been described in patients suffering from chronic inflammatory diseases [6], [7]. In mice, LPS-stimulated B cells express DAP12 in association with the immunoglobulin-like receptor II (MAIR-II) [8], [9]. Surprisingly, although the expression of ITAM-bearing adaptors is crucial to the expression of various activating immunoreceptors, some DAP12-negative T cells can express activating KIR [10]. These data suggest that an as yet unidentified adaptor molecule could associate with and stabilize cell surface expression of activating KIR in T cells that do not express DAP12.

The function of DAP12 is more complex than originally thought, as it can downregulate TLR-dependent responses in macrophages as well as CD16-dependent responses in NK cells [11], [12]. Similarly, DAP12 down-modulates the cytokine production by plasmacytoid dendritic cell (pDC) *in vivo* during murine cytomegalovirus infection [13]. Unraveling molecular mechanisms by which DAP12 can induce either activating or inhibiting signals will provide major informations on the fine tuning of immune responses.

Mutations in the human *DAP12* gene induce a rare pathology named polycystic lipomembraneous osteodysplasia with sclerosing encephalopathy (PLOSL), also known as Nasu-Hakola disease [14]. These patients do not present any obvious immunological defects, but are affected by severe bone and brain alterations. At early adulthood, first symptoms are pain and frequent fractures in the bone. The bone resorption is controlled by osteoclasts that derived from the monocytic lineage. *In vitro* the osteoclast differenciation is dramatically affected both in DAP12-deficient PLOSL patients and DAP12-deficient mice [15], [16], [17], [18]. Later PLOSL patients develop frontal lobe syndrome with a diffuse brain inflammation and dementia.

The combination of neurological, bone and inflammatory disorders which are associated with an alteration of DAP12 expression prompted us to generate a reagent compatible with diverse immunodetection procedures. Here, we describe the production and the characterization of a rat anti-human DAP12 monoclonal antibody. This antibody was used to determine DAP12 expression pattern in human peripheral blood leukocytes of normal subjects. We also investigated the DAP12 expression, in combination with the NK cell receptor repertoire, in systemic lupus erythematosus patients. Indeed, a reduced amount of NK cells, associated with altered functions and a down-modulation of DAP12 have been reported in this disease [19], [20], [21], [22].

## Results

### Characterization of a novel rat anti-human DAP12 monoclonal antibody

**Figure 1:**
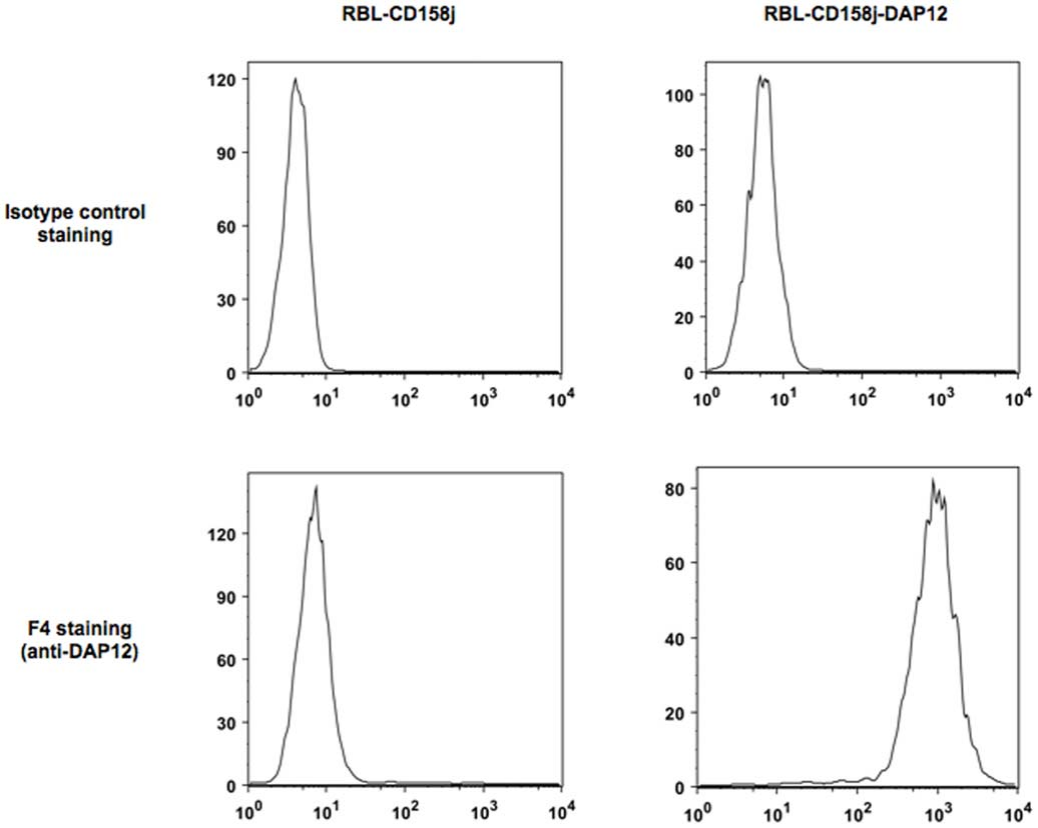
F4 MAb recognizes DAP12 antigen in transfected RBL cells. Stable transfectants of the rat basophilic leukemia cell line (RBL) have been described earlier [31]. In brief, RBL-CD158j and RBL-CD158j/DAP12 have been obtained by transfection with human cDNAs encoding CD158j alone or both CD158j and human DAP12, respectively. RBL cells were fixed by incubation with 2% paraformaldehyde at room temperature. Then the cells were permeabilized by Permwash treatment (Beckton Dickinson) before staining with 50 µg/ml irrelevant or F4 antibody on ice for 30 min. After three washes in cold PBS, the cells were incubated 1∶100 FITC-conjugated goat anti-rat immunoglobulins.

The rat H10E12F4 IgG1 (thereafter referred as to F4 mAb) was selected by its ability to bind specifically the DAP12 protein in an ELISA test (data not shown). To further analyze its specificity, a flow cytometry analysis of DAP12 expression was performed on RBL cells expressing DAP12 (RBL-CD158j/DAP12) or not (RBL-CD158j). As shown in figure 1, a positive staining by F4 antibody was only detectable in permeabilized DAP12-positive RBL cells. This result was confirmed using lentiviral transduction of human DAP12 cDNA both in DAP12-negative CD8+ T cells and DAP12-negative HEK cells (data not shown). This F4 mAb can be also used to detect DAP12 in Western-Blot as well as in immunohistochemistry assays (data not shown).

### DAP-12 expression pattern in healthy and systemic lupus erythematosus patients

**Figure 2:**
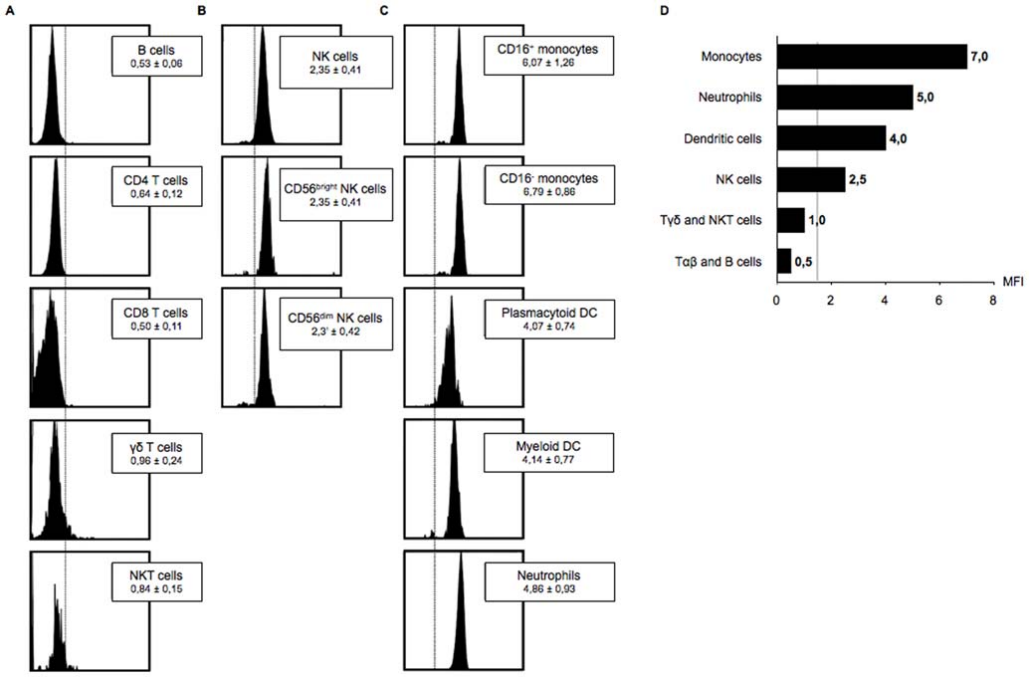
Analysis of DAP12 expression on leukocytes. Whole-blood samples from 10 healthy donors were collected and stained in a first step with several surface markers. The following mouse mAb from Immunotech-Beckman-Coulter were used: FITC-CD19 (J4.119), ECD-CD3 (UCHT1), FITC-CD16 (3G8), FITC-CD4 (13B8.2), FITC-CD8 (B9.11), FITC-CD33 (D3HL60.251), PECy7-CD14 (RMO52), FITC-TCR Vα_24_ (C15), PE-TCR panγδ (IMMU510), PECy7-CD56 (N901), PE-ILT3 ou PE-CD85k (ZM3.8). The blood was then treated with the IntraPrepTM permeabilization reagent and stained with DAP-12-Alexa 647-conjugated antibody (H10E12F4) (Immunotech-Beckman-Coulter). A. After gating lymphocyte population using SSC/FSC plot, T and B lymphocyte sub-populations were defined as follows: B lymphocytes are CD19^+^ cells, auxiliary T lymphocytes are CD3^+^/CD4^+^ cells, cytotoxic T lymphocytes are CD3^+^/CD8^+^ cells, γδT lymphocytes are TCRγδ-positive cells and NKT lymphocytes are CD56^+^/CD3^+^/Vα_24_^+^. B. After gating lymphocyte population using SSC/FSC plot, total NK cells are defined as CD3^−^/CD56^+^ cells and analyzed according to their level of CD56 expression. C. Monocytes were defined on the size/CD33^+^ gate as CD16^+^ or CD14^+^. Expression of DAP12 was analysed in these double-positive cells that can express or not the CD16 molecule. Dendritic cells were defined as ILT3^+^/CD14^dim to neg^CD16^neg^ within the lymphocyte and monocyte gate and further characterized as plasmacytoid CD33^low^ DC and conventional CD33^high^ DC. Neutrophils were defined on the size/CD16^+^ gate. D. Schematic representation of the expression of DAP12 by leukocyte subsets from healthy individuals.

Since the original description of DAP12 expression in NK cells, a broader expression among other immune cells has also been found in mouse and human, both in myeloid and lymphoid cells, and many DAP12-associated activating membrane receptors have been described. However, data about DAP12 expression in humans relies on Western blot and microarrays studies. Thus, the F4 mAb was used to provide additional data about the expression of the polypeptide DAP12 in leukocytes from healthy individuals. Whole blood sample from healthy donors were stained in a first step by lymphocytes surface markers (CD4, CD8, CD19, CD56, CD3, Vα_24_, Vβ_12_, TCRγδ). Then the cells were permeabilized and stained by Alexa 647-conjugated F4 antibody. DAP12 expression was barely detected in CD8^+^ T lymphocytes, CD4^+^ T lymphocytes, CD19^+^ B lymphocytes, γδ^+^ T lymphocytes and Vα_24_ NKT lymphocytes (Figure 2A). By contrast DAP12 expression was detected in the NK cell population with no difference between CD56^bright^ and CD56^dim^ sub-populations (Figure 2B).

The expression of DAP12 in other cells involved in innate immunity and expressing several DAP12-associated receptors was then evaluated (Figure 2C). Among these cells, monocytes express TREM-1, SIRP-β, MDL-1 and IREM-2 receptors. Thus, F4 staining on distinct monocyte sub-populations that co-expressed CD14 and CD33 molecules was analysed. A strong staining of both CD16^+^ and CD16^−^ monocyte subsets was observed. Dendritic cells (DC) also express several DAP12-associated receptors, such as PIRL-β, SIRP-β, IREM-2 and TREM-2. DC were defined as ILT-3^+^/CD14^+^/CD16^−^ cells. DAP12 was highly expressed both in plasmocytoid (CD33^low^) and conventional (CD33^high^) DC sub-populations. Finally neutrophils are DAP12-positive cells.

Since it was previously described a lower NK cell number in SLE patients, we have quantified and compared the leukocyte subsets from SLE patients and control individuals. NK absolute counts and percentage of total lymphocyte were first compared to our large control group of 92 healthy donors. The mean of NK absolute counts was significantly decreased in SLE patients compared to controls (106/µl±15 versus 259/µl±14, p<0.0001) (Table 1). The NK cell percentage of total lymphocytes was also significantly decreased in SLE patients compared to this control group (7.81%±1.19 versus 12.01%±0.59, p = 0.011). However, when compared to the control group of 10 healthy donors, used for the NK cell phenotype analysis, there was only a trend toward a decrease in NK cell percentage in SLE patients that did not reach significance (7.81%±1.19 in SLE patients compared to 11.59%±2.25 in controls, p = 0.12). There is no significant change in the proportion of CD56^dim^ and CD56^bright^ subsets between the two groups (Table 2).

**Table 1:** Number of NK cells in SLE and control patients.

	Controls (n = 92)	SLE (n = 13)
**Absolute number**	259±14/µl	106±15/µl
**%**	12.01±0.59	7.81±4.30

Absolute numbers and percentage of NK cells among total lymphocytes are indicated for the control group (n = 92) and the SLE group of 13 patients.

**Table 2:** Percentage of leukocytes subsets and DAP12 expression in SLE and control patients.

		% of DAP12-positive cells		MFI of DAP12 staining	
		Controls (n = 10)	SLE (n = 13)	Controls (n = 10)	SLE (n = 13)
**NK**		11.59±7.13	7.81±4.3	2.91±0.64	3.67±1.05
	*CD56^bright^*	*3.77±6.47*	*11.53±10.63*	*6.37±3.76*	*6.68±3.36*
	*CD56^dim^*	*89.56±13.38*	*87.30±11.09*	*2.47±0.48*	*2.86±0.78*
**DC**	Plasmacytoid DC	0.24±0.07	**0.16±0.08**	4.06±0.74	3.62±1.46
	Conventional DC	0.32±0.09	**0.19±0.09**	4.14±0.77	4.29±0.64
**Monocytes**	CD33^+^ CD14^+^	6.92±1.83	7.21±2.07	7.22±1.17	6.72±0.99
	CD33^+^ CD16^+^	0.87±0.36	1.10±0.96	6.06±1.26	6.62±1.32
**Neutrophils**		47.79±8.95	55.57±10.86	4.86±1.40	4.80±0.93

Both percentage of NK cells among total lymphocytes and percentage of the CD56^bright^ and CD56^dim^ subsets among NK cells are indicated. The percentage of dendritic cells, monocytes and neutrophils among the leukocytes are given. Statistically significant values are shown in bold.

The quantification of other leukocytes subsets has shown that the percentage of circulating DC was significantly decreased in SLE patients compared to controls, considering both plasmacytoid DC (0.16±0.08 versus 0.24±0.074, p = 0.04) or conventional DC (0.19±0.09 versus 0.32±0.09, p = 0.003) (Table 2). On the contrary, the percentages of neutrophils or monocytes were not different in SLE patients compared to controls.

It was previously described by western-blot analysis a down-regulation of DAP12 expression in NK cells from SLE patients. In addition, DAP12 is also expressed by other leukocytes such as DC which have been clearly involved in lupus physiopathogeny. This prompted us to extend on leukocyte subsets the analysis of DAP12 expression in SLE. The number of DAP12-positive NK cells was slightly decreased compared to our control group of healthy subject (96.67%±2.02 versus 97.66%±3.23, p = 0.02) (data not shown). Conversely, there is no significant modulation of the MFI for DAP12 expression by NK cells (Table 2). The expression of DAP12 in DCs, monocytes and neutrophils are similar in the two groups (Table 2).

### NK cell receptor repertoire of systemic lupus erythematosus patients

It has been shown that NK cell functions are altered in SLE patients. A modulation in the expression of activatory and inhibitory NK cell receptors, implicated in the tuning of NK cell activation, could be associated with a decrease in NK cell functions. Moreover DAP12 is crucial to signalling of many NK cell activatory receptors.

**Figure 3:**
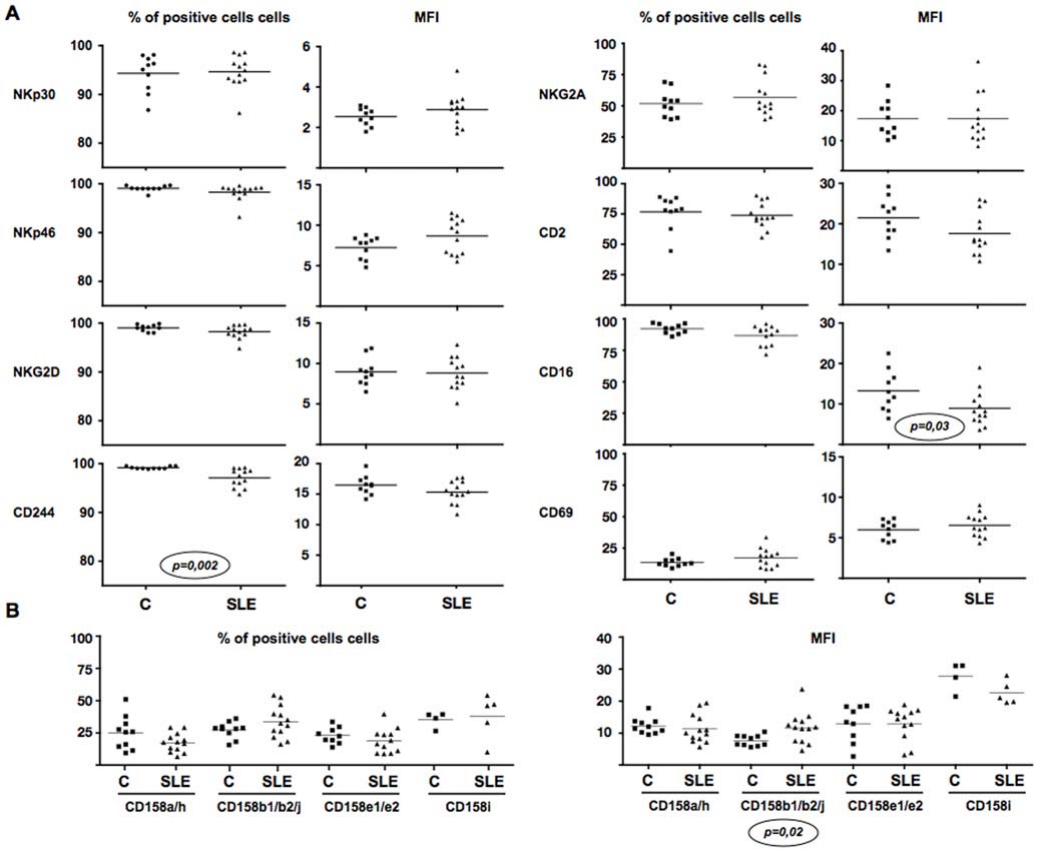
NK cell receptors percentage and MFI in SLE patients and controls. Cell surface expression of indicated molecules on NK cells within peripheral blood lymphocytes from controls (squares) and SLE patients (triangles) are given as percentage of total NK cells and MFI. Horizontal bars indicate median values. The *p* value is mentioned above a given NK cell subset when a statistically significant difference is observed between the two groups.

Phenotypical characterization of NK cells was performed by analyzing the expression of many receptors such as NKp46, NKp30, NKp44, NKG2D, CD244, CD2, CD16, CD69, CD159a, CD158a/h, CD158b1/b2/j, CD158e1/e2, CD158i and NKG2A. The results show only minor phenotypical changes between SLE patients and controls (figure 3). There is a significant decrease of CD16 MFI (p = 0.03) and of the proportion of CD244^+^ NK cells (p = 0.002) associated with an increase of the CD158 b1/b2/j MFI (p = 0.02) in the SLE group. No difference for the expressions of NKp46, NKp30, NKp44, CD2, CD69, NKG2A, CD158a/h, CD158e1/e2 and CD158i was observed in SLE patients compared to controls.

## Discussion

We have generated a rat monoclonal antibody (F4) directed against human DAP12 for which the specificity was validated in three independents transfection models. This anti-human DAP12 monoclonal antibody represents a powerful tool to investigate DAP12 expression in patients.

This report is the first description of basal expression of DAP12 protein in distinct human leukocyte subpopulations from healthy individuals. These results validate the restricted expression of DAP12 in cells involved in innate immunity (Figure 2D) and are consistent with public databases of transcript pattern of DAP12 (http://symatlas.gnf.org) [23].

In some individuals, a dull staining with the F4 mAb was detected. This F4 staining was abrogated upon preincubation of F4 antibody with the GST-DAP12 immunogen and was unchanged after preincubation of F4 antibody with an irrelevant GST protein, suggesting a specific but weak binding (data not shown). Then, we hypothesized that alternative transcripts of DAP12, that are not detected by classical RT-PCR, are recognized by the F4 mAb. To address this issue, specific primers of DAP12 were designed to amplify from exon 1 to 3, 1 to 4, 1 to 5 and 3 to 5. Using these primers, no transcripts were detected in cells known as DAP12 negative (data not shown). The F4 mAb could also cross-react with an as yet unknown protein. Since F4 mAb does not recognize mouse DAP12, we aligned proteic sequence of human and mouse intracellular domains of DAP12 and we identified species specific amino acid sequences. Most of gaps were found in the aminoterminal part of the intracellular domain. This region was blasted using NCBI server, but no significant hit was found. At this time, we are not able to explain the dull staining observed in some DAP12-negative cells. We cannot formally exclude the existence of another adaptor that could be recognized by F4 mAb with a weaker affinity compared to DAP12.

To conclude, the rat IgG1monoclonal antibody F4 allows an efficient detection of DAP12 expression in monocytes, dendritic cells, neutrophils and natural killer cells. Indeed, in some inflammatory conditions, it would be a useful reagent to monitor DAP12 expression in B and T cell populations.

The circulating NK cells from our cohort of SLE patients presented only mild phenotypical changes when compared to NK cells from controls. An increase of the CD56^bright^ subset has been reported in SLE patients [24]. This NK cell subset is of interest in SLE because it represents the main NK cells found in secondary lymphoid tissues and inflamed tissues. We also report a weak increase of the CD56^bright^ subset, however this change is not significant due to the great interindividual variability. This discrepancy could be explained by the low activity index of SLE in our cohort.

Activation of NK cells is finely tuned by the expression of MHC class I NK cell receptors. Moreover susceptibility to some autoimmune and inflammatory diseases have been genetically linked to these receptors [25]. In our study, the levels of NKG2A (CD159a), CD158a/h, CD158e1/e2, CD158i expression by NK cells from SLE patients were in the normal range with the exception of the CD158b1/b2/j receptors for which an increased MFI is observed. However, the percentage of CD158b1/b2/j^+^ NK cells was similar in SLE patients and controls.

Along the same line, the abnormal expression of the Natural Cytotoxicity Receptors (NCR) reported in primary Sjögren syndrome was proposed to explain the decrease of NK cell cytotoxity in this autoimmune disease [26]. By contrast to these results, the expression of NKp44 (data not shown), NKp46 and NKp30 were not significantly different from controls. We also investigated the expression of CD244, CD16 and CD2. Although the percentage of NK cells expressing CD244 was slightly decreased, there is no difference in the MFI. Moreover, while the MFI of the CD16 staining was also slightly decreased, there is no modification of the percentage of CD16^+^ NK cells. At last, the expression of CD2 is identical in the two groups. Thus, altogether these results show the absence of a major modulation of the expression of NK cell receptors in our cohort of SLE patients.

Since the DAP12 adaptor molecule is used by many receptors to transduce activating signals, its expression was analysed in distinct leukocyte subpopulations by flow cytometry using the F4 mAb. No significant changes were found both in NK cells and leukocyte subpopulations from SLE patients compared to controls. Only few NK cells are DAP12-negative in SLE patients and the percentage of expression are very close to controls. Contrary to our results, *Toyabe et al*. have reported a decreased expression of DAP12 in NK cells from SLE patients by using western blot analysis with a rabbit anti-human DAP12 antibody [21]. Similarly to our study, their patients were mostly untreated and they displayed a relatively low SLEDAI score, suggesting that this discrepancy is not related to patient characteristics but rather to the methods used for DAP12 detection in NK cells.

To conclude, our results confirm that the basal expression of DAP12 is restricted to cells of innate immunity in healthy individuals. In addition, this study shows that the functional deficiency of NK cell cytotoxicity in SLE patients is not related to an abnormal expression of the NK cell receptors or DAP12 protein, as it has been previously shown with NCRs in acute myelogenous leukaemia [27].

## Materials and Methods

### Generation and characterization of a novel mAb directed against human DAP12

Hybridomas were generated by fusion of splenocytes from an immunized rat with the Ag8-X63 plasmocytoma. Rats Lou (Harlan) were immunized with a GST-DAP12 protein containing the intracellular moiety of human DAP12 fused to the GST protein. The coding sequence of the intracellular part of human DAP12 was obtained by PCR using specific primers (5′ primer, 5′GGGATCCCGGCTGGTCCCT-3′ and 3′ primer, 5′-CGAATTCTGTCATGATTCGGGC-3′) and cloned in BamHI and EcoRI restriction sites of pGEX-4-T1 vector (GE Healthcare). Bacteria transformation and fusion protein production and purification were performed according to manufacturer's instructions (GE Healthcare). Rats were subcutaneously immunized at 3-weeks intervals with 100 µg of purified GST-DAP12 solution emulsified in an equal volume of Freund's adjuvant (complete for the first injection and uncomplete for the following boosts). The fusion protocol was previously described [28]. Briefly, splenocytes from immunized rats were fused with Ag8-X63 plasmocytoma cells. Ten days after the fusion, the presence of specific antibodies in supernatants of hybridoma cultures was evaluated by ELISA. Positive hybridomas were cloned twice by limiting dilution in 96-well culture plates. Purification of the monoclonal antibody was performed by protein G-sepharose affinity chromatography.

### Patients

Patients enrolled in the study (n = 13) were recruited from our Department of Internal Medicine. They presented with SLE and fulfilled the American Rheumatism Association 1982 revised criteria, except 2 patients presenting with only 3 criteria and thus considered as lupus-like syndrome. Characteristics of the patients and ongoing treatments are summarized in Table 3. Disease activity was assessed by a modified SLE disease activity index (SLEDAI) score [29]. 10 healthy volunteers (controls) were recruited from the CPCET (Centre de Pharmacologie Clinique et d'Evaluations Thérapeutiques). CPCET is an official facility from our hospital where healthy volunteers are enrolled in studies after written informed consent is obtained in accordance with ethics and law. For NK cell absolute numbers and percentage of total lymphocytes, additional statistical comparison was made with values obtained in a previously reported large control cohort of healthy subjects [30]. This study did not require the approvement of an ethical committee as it was performed without any supplemental blood prelevement. The analysis were performed on blood samples used for routine analysis during the follow-up. A patient written informed consent was obtained in accordance with the French law.

**Table 3:** Patients characteristics.

N	Age	Sex	WBC	Ly	NK	Treatment	ANA titer	Type	dsDNA titer	ACR
1	45	f	2340	790	79	MMF+P	800	SSA/SSB	216	7
2	27	m	9490	4600	170	H+P	800	Sm/RNP	2	5
3	27	m	5970	1740	158	H+P	800	RNP	31	5
4	26	f	4750	1540	92	H	400	SSA	6	3
5	34	f	6440	2080	62	H	600	-	2	4
6	28	f	6700	2180	218	H+P	600	SSA	4	6
7	40	f	3390	790	95	H+P	800	RNP/SSA	199	5
8	32	f	5890	1370	96	H+P	100	SSA	69	4
9	29	f	6790	710	35	H+P	400	-	26	4
10	43	f	4580	1140	80	P	800	SSA/SSB	31	4
11	28	f	2990	1290	51	P	200	-	1	4
12	48	f	7790	1830	165	P	800	RNP	2	3
13	38	f	2370	840	79	none	800	SSA/SSB	2	4

Quantification of total White blood cells (WBC), total lymphocytes (Ly) and NK cells are indicated in cells per µl. Treatment for each patient is shown: MMF for mycophenolate mofetil, H for hydroxychloroquine and P for prednisone (at time of analysis all patients received ≤10 mg/day of prednisone). Main biological characteristics of autoantibodies are shown: antinuclear antibodies (ANA) titer, type of ANA and titer of anti-dsDNA autoantibodies (N<7). The number of ACR criterias for each patient is indicated.

### MAb and Flow cytometry

The following mouse mAbs were used: ECD-CD3 (UCHT1), FITC-CD16 (3G8), FITC-CD4 (13B8.2), FITC-CD8 (B9.11), FITC-CD33 (D3HL60.251), PECy7-CD56 (N901), PE-ILT3 ou PE-CD85k (ZM3.8), PE-NKG2D (ON72), PE-NKp30 (Z25), PE-NKp44 (Z231), PE-NKp46 (Bab281), PE-CD159a/NKG2A (Z199), PE-CD158a/h (EB6B), PE-CD158b1/b2/j (GL183), PE-CD158e1/e2 (Z27.3.7), PE-CD158i (FES172), PE-CD69 (TP1.55.3), PE-CD2 (39C1.5), PE-CD244 (C1.7) and DAP-12 Alexa 647 (H10E12F4), (all from Beckman Coulter). NK cells were defined as the CD3^−^CD56^+^ cells within the lymphocyte size/structure gate. Dendritic cells were defined as ILT3^+^/CD14^dim to neg^CD16^neg^ within the lymphocyte and monocyte gate and further characterized as plasmacytoid DC CD33^low^ and conventional DC CD33^high^. Monocytes were defined on the size/CD33^+^ gate as CD16^+^ or CD14^+^. Neutrophils were defined on the size/CD16^+^ gate.

Whole blood samples from donors were collected and stained in a first step with several surface markers. The blood was then treated with a permeabilization reagent (IntraPrep™ permeabilization reagent IM2388) and stained with DAP-12 Alexa-647 conjugated antibody. All samples were analyzed on a Cytomics FC500 flow cytometer using CXP software (Beckman Coulter, Miami, US).

### Statistical analysis

Graphic representation and statistical analysis of NK cell phenotype were obtained using Graphpad Prism software (San Diego, CA). Comparison of distributions was performed using the Mann-Whitney U-tests. Results were considered as significant with a p<0.05.
